# Radiographic identification of symptomless mandibular third molars without clinical pericoronitis

**DOI:** 10.1007/s00784-024-05953-3

**Published:** 2024-09-30

**Authors:** Tommi Vesala, Irja Ventä, Johanna Snäll, Marja Ekholm

**Affiliations:** 1 Department of Oral and Maxillofacial Diseases, Faculty of Medicine, University of Helsinki, P.O. Box 41, Helsinki, FI-00014 Finland; 2 Department of Oral and Maxillofacial Diseases, Helsinki University Hospital, Helsinki, Finland

**Keywords:** Molar, third, Radiography, panoramic, Pericoronitis, Tooth eruption, Diagnosis, oral

## Abstract

**Objectives:**

The aim was to identify radiographic characteristics of mandibular third molars in young adults without symptoms or clinical signs of pericoronal infection.

**Materials and methods:**

An existing cross-sectional material, including records from clinical oral examination and panoramic radiographs (PANs) of university students, was submitted to retrospective analysis. The outcome variable was a symptomless and clinically pericoronitis-free mandibular third molar. Predictor variables for the third molar were clinical eruption level, pathological signs in the follicle, marginal bone level, radiographic depth in bone, inclination, stage of root development, and available space for eruption. Statistics included χ^2^ and Mann-Whitney U tests.

**Results:**

Analysis included 345 mandibular third molars in 189 participants (20% men, 80% women; mean age 20.7 years; SD ± 0.6). Symptomless and clinically pericoronitis-free mandibular third molars were characterized as follows: clinically unerupted in 78% of teeth, associated with reduced marginal bone level in 70%, located deeper in the bone in 87%, mesially inclined in 73%, and stage of root development incomplete in 68% (*p* ≤ 0.001 for all).

**Conclusions:**

Radiographic characteristics of symptomless mandibular third molars without clinical pericoronitis in young adults can be assessed from a PAN with 68–87% certainty.

**Clinical relevance:**

These findings may prove useful when trying to exclude non-pathological mandibular third molars from diseased teeth.

## Introduction

One of the common radiographs interpreted by oral radiologists is dental panoramic radiography, which is also the most frequent choice for third molar imaging [1, 2]. Radiologists are occasionally obliged to make statements about panoramic radiographs (PANs) accompanied by incomplete or no clinical information. Based on a radiograph alone, it is challenging to determine whether third molars are potential infection foci. Therefore, the radiologist needs information on the clinical situation. However, it would be useful if one could identify from a PAN some characteristics typical of symptomless third molars.

Among the common diagnoses to extract third molars are pericoronitis, caries, and impaction [3, 4]. Pericoronitis is a clinical diagnosis associated mostly with mandibular third molars, yet signs of pericoronitis may be visible in radiographs. When the tooth has perforated the marginal bone cortex and gingiva, the integrity of the dental follicle is breached, and subsequently, the third molar is exposed to oral bacterial flora. Hence, partial eruption is a widely recognized predisposing factor for the development of pericoronitis [5–8].

A correlation between pericoronitis and position of the mandibular third molar has been widely reported [8]. However, less scientific evidence is available on radiographic characteristics of mandibular third molars without clinical signs of pericoronal infection in symptomless persons. According to a Turkish study on 342 patients, completely unerupted mandibular third molars are less likely to have symptoms or pericoronitis than partially or fully erupted ones [9]. Nevertheless, it is important to note that lack of symptoms does not equate to lack of pathology [10].

The aim of this study was to determine radiographic characteristics of mandibular third molars in persons without symptoms or clinical signs of pericoronal infection. The hypothesis was that typical radiographic characteristics of such teeth can be identified.

## Materials and methods

### Study design

A retrospective study on existing cross-sectional data was designed to evaluate radiographic characteristics in clinically pericoronitis-free mandibular third molars of symptomless young adults. The data were collected at the Finnish Student Health Service (FSHS), Helsinki, Finland in 2002 [11, 12]. All first-year students at the University of Helsinki were routinely invited to participate in a free oral health examination at the FSHS. Of these, a cohort of 277 students was selected based on their being born in Helsinki in 1981 or 1982 and living in Helsinki at the beginning of their studies. Students of the cohort completed a questionnaire and after the clinical oral examination, they were offered a possibility to participate voluntarily in the radiography. Participants within a narrow age range and with similar backgrounds, including birthplace and current place of residence, were selected to minimize potential bias of the material.

**Fig. 1:**
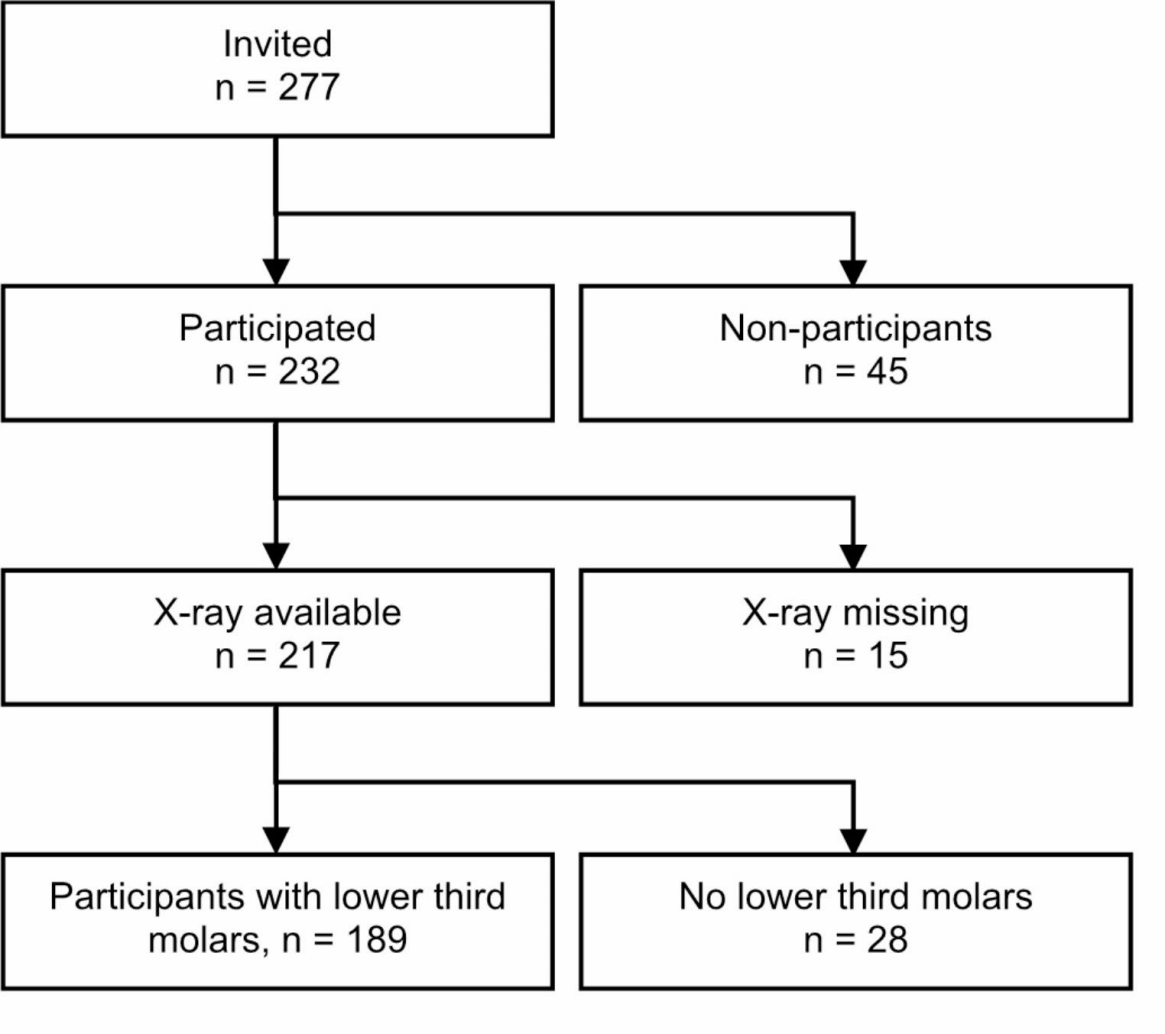
Flow diagram of included and excluded participants

The criteria for exclusion from the study were a PAN not being available for the present analysis and no mandibular third molars visible on the PAN. Of the 277 invited students, 45 (16%) were excluded for not participating in the clinical oral examination (Fig. 1). Another 16% were excluded for missing PANs or mandibular third molars. A missing data analysis of included and excluded participants showed that they did not differ by sex (χ^2^ = 1.26; df = 1; *p* = 0.261) or age (Mann-Whitney U = 4160; *p* = 0.808).

### Study variables

The material included responses to the questionnaire on symptoms of third molars, results of the clinical oral examination, and PANs. Age and sex of participants were recorded. Clinical features of the mandibular third molars included identification of the tooth, its clinical stage of eruption, and signs of clinical pericoronitis (Table 1).

**Table 1:** Definitions of clinical study variables

Variable		Category	Definition
Questionnaire			
	Symptoms in third molars	No	I have had no pain or symptoms
		Yes	My wisdom teeth have caused pain or symptoms
Clinical features			
	Clinical stage of eruption	Unerupted	Clinically invisible and cannot be probed
		Connected to the oral cavity	Crown completely or partially visible or can be felt with a probe in the distal pocket of the second molar
	Pericoronitis	Yes	Diagnosed pericoronitis in clinical oral examination
		No	No clinical signs of inflammation in the soft tissues around the third molar

Radiological variables of the third molars comprised pathological signs in the follicle, marginal bone level on the distal surface of mandibular second molar, depth of a tooth in the alveolar bone, inclination, stage of root development, and mesiodistal space for eruption (Table 2). Mesiodistal space for eruption was evaluated according to the Pell and Gregory classification, as described elsewhere [5].

**Table 2:** Definitions of radiographic variables of the mandibular third molars

Variable	Category	Definition
Signs in the follicle	Pathological	Margin of the follicle uneven, sclerotic bone formation around the crown, or the greatest width of the follicle > 2.5 mm
Marginal bone level	Reduced	At the cervical, middle, or apical level of the second molar
Alveolar bone depth of the most cranial point of tooth [12]	A	Below the marginal cortex
	B	In the marginal cortex, but not perforating it
	C	Has perforated the marginal cortex: location below or at cementoenamel junction of the second molar
	D	Has perforated the marginal cortex: location between cementoenamel junction of the second molar and occlusal surface
	E	Located at the occlusal surface
Inclination	Vertical	0° ‒ 10°
	Distoangular	-1° ‒ -70°
	Mesioangular	(+ 11° ‒ +70°)
	Horizontal	Mesiohorizontal ( > + 70°) or transversal
Stage of root development	Incomplete	Apices of roots not closed
	Finished	Apices closed
Mesiodistal space for eruption	Class I	Sufficient space for eruption
	Class II	Not sufficient space for eruption
	Class III	No space for eruption

The outcome variable was a symptomless mandibular third molar without clinical signs of pericoronal disease. Predictor variables were the clinical stage of eruption and the six radiographic characteristics of mandibular third molars on PANs.

### Radiological examination

PANs were taken with Planmeca Promax 2D (Helsinki, Finland) with exposure values of 64‒68 kV voltage, 6.3‒10 mA current, and 15.8 s time. The PANs were analysed at the facilities of the FSHS by one of the authors. After analysis of all radiographs, 11% (*n* = 23) of randomly chosen radiographs were analysed a second time after two weeks to obtain an estimate of intra-examiner reproducibility.

### Ethical considerations

The FSHS Institutional Review Board approved the clinical and radiographic examinations in 2002. The oral health examinations adhered to the Declaration of Helsinki guidelines, and each student participated voluntarily after signing an informed consent. Following the European Commission guidelines for radiation protection, it is deemed unacceptable practice to conduct routine radiography without the patient’s history and a clinical examination [1]. Therefore, an existing radiographic material was repurposed for the current analysis. The Finnish Social and Health Data Permit Authority (Findata) approved the secondary utilization of this health care data (THL/4680/14.02.00/2020). The FSHS also granted permission to employ the existing material for the present study. For reasons of data protection, results were not presented if the frequencies were less than 5, and therefore, in the analysis of variables some combinations of categories were made (marginal bone level and depth in alveolar bone).

### Statistical analysis

Mandibular third molar was the unit of observation. In the analysis, characteristics of third molars were cross-tabulated according to symptomless and symptomatic persons. Differences between subgroups were examined using χ^2^ test for frequencies and Mann-Whitney U test for means of independent groups. The significance level was set at *p* < 0.05. SPSS Statistics version 27 (IBM Corporation, Armonk, NY, USA) was used in the analyses.

## Results

The number of participants included in the analyses was 189 (20% men, 80% women). Their mean age was 20.7 years (standard deviation (SD) ± 0.6 years, range 19.7–21.7 years). These 189 persons had 345 mandibular third molars.

Regarding intra-examiner reliability of the radiographic characteristics, the kappa values were 0.95 for marginal bone level, 0.95 for depth of the tooth in the bone, 0.94 for stage of root development, and 1.00 for other characteristics. A value of 0.81 or above indicated almost perfect agreement.

According to the questionnaire and the clinical oral examination, 58% (*n* = 110) of the participants were symptomless and had no clinical signs of pericoronal infection in their mandibular third molars. No significant difference existed between men (68%) and women (56%) (χ^2^ = 2.04; df = 1; *p* = 0.153). In this symptomless group, the number of mandibular third molars was 203 (59% of all 345 mandibular third molars).

An analysis of mandibular third molars according to presence or absence of clinically detected pericoronitis and symptoms is presented in Table 3. Symptomless mandibular third molars without clinical pericoronitis were most likely clinically unerupted (78%; χ^2^  = 59.44; df = 1; *p* < 0.001), and radiographically, were associated with reduced marginal bone level (all reduced levels combined: 70%; χ^2^  = 12.62; df = 1; *p* < 0.001), located deeper in the bone (classes A, B, and C: 87%; χ^2^  = 36.26; df = 2; *p* < 0.001), mesioangularly inclined (75%; χ^2^  = 48.44; df = 3; *p* < 0.001), and had incomplete root development (68%; χ^2^  = 10.67; df = 1; *p* = 0.001). Pathological signs in the follicle were not associated with clinical signs and symptoms (χ^2^  = 3.81; df = 1; *p =* 0.051). Mesiodistal space for eruption also was not a significant predictor.

**Table 3:** Analysis of 345 mandibular third molars according to presence or absence of clinically detected pericoronitis and symptoms

Variable		Third molars in persons with		
		No symptoms or clinical pericoronitis *n* = 203	Symptoms or clinical pericoronitis *n* = 142	*p* value^a^
		n (%)	n (%)	
Clinical				
Clinical stage of eruption	Unerupted	144 (78)	41 (22)	< 0.001
	Connected to oral cavity	59 (37)	101 (63)	
Radiological				
Changes in the follicle	No	159 (62)	98 (38)	0.051
	Yes	44 (50)	44 (50)	
Marginal bone level behind the second molar (*n* = 342)^b^	No reduction	101 (51)	97 (49)	< 0.001
	Reduced to cervical level	69 (66)	36 (34)	
	Reduced to middle or apical level	32 (82)	7 (18)	
Depth of tooth in alveolar bone [12]	Below or at CE junction of the second molar (A, B, C)	33 (87)	5 (13)	< 0.001
	Between CE junction of the second molar and occlusal surface (D)	109 (68)	51 (32)	
	At the occlusal surface (E)	61 (41)	86 (59)	
Inclination	Vertical	43 (50)	43 (50)	< 0.001
	Distoangular	17 (27)	47 (73)	
	Mesioangular	132 (75)	45 (25)	
	Horizontal	11 (61)	7 (39)	
Stage of root development	Incomplete	112 (68)	53 (32)	0.001
	Finished	91 (51)	89 (49)	
Mesiodistal space for eruption	Sufficient space for eruption (Class I)	36 (57)	27 (43)	0.443
	Not sufficient space for eruption (Class II)	141 (58)	103 (42)	
	No space for eruption (Class III)	26 (68)	12 (32)	

^a^Pearson χ^2^-test

^b^In three cases, the level of bone could not be determined

CE: cementoenamel

## Discussion

**Fig. 2:**
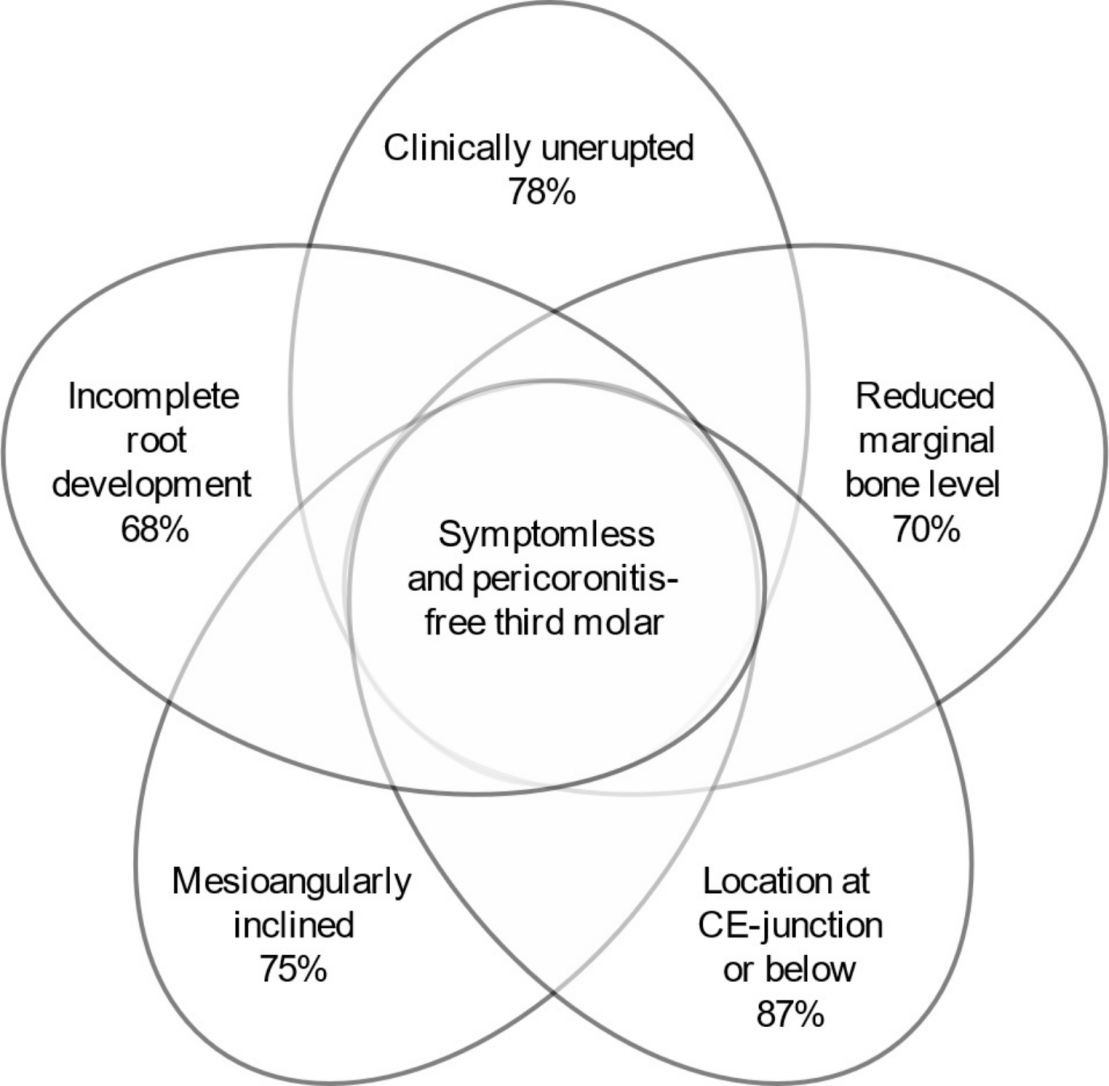
Common characteristics of a symptomless mandibular third molar without clinical pericoronitis. Numbers below each variable indicate the proportion (%) of teeth with the characteristic. CE: cementoenamel

The purpose of this study was to identify radiographic characteristics of mandibular third molars without symptoms or clinical pericoronitis. Such teeth were radiographically most likely associated with reduced marginal bone level on the distal surface of the mandibular second molar, deep in bone or only just perforating the cortex, mesioangularly inclined, and had incomplete root development (Fig. 2). In addition, symptomless teeth were most likely clinically unerupted.

The most surprising finding was the significant association between reduced marginal bone level on the distal surface of the second molar and the lack of symptoms and clinical pericoronitis. Moreover, the greater the reduction in marginal bone level, the fewer the symptoms or clinical pericoronitis. Marginal bone loss is typically considered a pathological finding and often associated with mesial inclination [5, 13, 14]. The present finding may be explained by the narrow age range (19.7–21.7 years) of the sample, and thus, the eruption phase of the third molar being underway [15]. Therefore, the reduced alveolar bone level may be more related to the eruption process and bone remodelling around the dental follicle [16] than to current pathology. This question is examined in a Japanese study on 241 patients with mesioangularly impacted mandibular third molars [17]. That study concluded that bone resorption was not related to acute pericoronitis in young adults (18‒22 years) but was related in older patients (≥ 41 years). Still, it is important to note that third molars with reduced bone level and unable to erupt may be at risk for periodontal pathology if a connection to the oral cavity is later established [18–20].

The present finding on the clinical stage of eruption of third molars is in line with earlier study in that the majority of unerupted teeth are symptomless and without clinical pericoronitis [9]. This is also evident in a British study with the UK strategy to remove only symptomatic third molars, as 97% of removed teeth were partially or totally erupted [6]. The present variable of clinical stage of eruption was similar to the radiographic depth in bone; superficially located teeth were often associated with symptoms or pericoronitis. This is consistent with a Spanish study on patients undergoing extraction of mandibular third molars, where the occurrence of pericoronitis was lower the deeper the tooth was located [5].

Regarding inclinations of teeth, mesioangular third molars were more likely symptomless and pericoronitis-free than vertical or distoangular teeth. The finding of distoangular inclination as the most prone to pericoronitis has also been reported earlier [7]. A recent meta-analysis notes similarly that vertical teeth are most frequently associated with pericoronitis but horizontal teeth least frequently [8]. However, in that meta-analysis, most studies included patients referred to third molar extraction, while the material of the present study was gathered through a routine oral examination. Therefore, in the earlier studies, inclinations in symptomless and symptomatic persons are rarely compared, and consequently, certain inclinations may be overrepresented. Furthermore, in most of the studies of the above-mentioned meta-analysis, the inclination was assessed according to Winter’s classification, which differs slightly from the present study in the limit values of vertical and distoangular inclinations.

Incomplete root development was associated with a lack of symptoms and clinical pericoronitis. This is surely related to the development of the third molar, as teeth with incomplete root formation are more likely unerupted. A similar finding was made in a Danish follow-up study on 132 adolescents, where the root development of erupted third molars by the age of 20 was ahead of that of impacted ones [21].

Radiographic changes in the follicle occurred at the same rate in the symptomless and symptomatic groups, and thus, poorly predicted clinical status. Radiographic assessment of the pericoronal space as normal or pathological is complicated. In the literature, pericoronal space < 2.5–3 mm is often considered to be normal [22–24]. However, in studies on patients referred to extraction of impacted third molars with < 2.4–2.5 mm pericoronal space, pathological changes in dental follicles were observed between 46% and 58.5% of the teeth in histopathological examination [24, 25]. This highlights the complexity of assessing pericoronal pathology based on radiographic changes in the follicle, increasing the challenge for radiologists when making statements about PANs with limited clinical information.

The present findings on the identification of symptomless mandibular third molars without clinical pericoronitis are likely to be applicable to all young adults when interpreting a PAN of third molars. The age range in this study corresponded to the typical age of third molar eruption. A strength of this study was that the participants were not patients referred to extraction of third molars but regular students who participated voluntarily in a routine oral health examination. Thus, the volume of symptoms and pathology of present third molars were not as prominent as in studies on patients.

A limitation of the present material was that although the presence of third molars was probed, the probing depths distal to second molars and around third molars were not obtained. Clinical probing depths combined with radiographic bone level data could have given more detailed knowledge of the current periodontal status of third molars. Another limitation was that the material was over 20 years old. This was because, according to the European Commission guidelines for radiation protection, it would be unacceptable to conduct a similar study solely for third molars [1]. Thus, an existing radiographic material was utilised for the current analysis. A third limitation was that the participants were university students from the capital of the country. They may thus have had better access to dental care, including prevention of third molar pathology, than the rest of the same-aged population. However, the narrow age range and similar background homogenized the material. A fourth limitation was the sex distribution, which was female-dominated. This is explained by 64% of bachelor’s degree students and 68% of master’s degree students at the University of Helsinki being women. Furthermore, women were more active than men in participating in the oral health examination (88% vs. 74%) [12].

The clinical relevance of the findings lies in situations where a patient has an ambiguous infection and infection foci are searched from the head and neck areas. In such cases, the initial assessment may be based solely on radiological findings. As the third molar is a potential focus of infection, the present findings may prove useful when trying to exclude non-pathological mandibular third molars from diseased teeth. The findings may also be useful when a radiologist is writing a statement, an expert body is making an insurance judgement, or in a consultation at the request of other clinicians.

## Conclusion

Mandibular third molars without symptoms or clinical pericoronitis in 21-year-old adults can be assessed from a PAN with 68–87% certainty. The best predictor was the location deep in the bone or only just perforating the cortex, followed by mesioangular inclination, reduced marginal bone level on the distal surface of the second molar, and incomplete root development in this order of decreasing certainty. However, radiographic changes in the follicle were unreliable predictors.

## Data Availability

No datasets were generated for public use during the current study.
